# The Highly Conserved Codon following the Slippery Sequence Supports −1 Frameshift Efficiency at the HIV-1 Frameshift Site

**DOI:** 10.1371/journal.pone.0122176

**Published:** 2015-03-25

**Authors:** Suneeth F. Mathew, Caillan Crowe-McAuliffe, Ryan Graves, Tony S. Cardno, Cushla McKinney, Elizabeth S. Poole, Warren P. Tate

**Affiliations:** Department of Biochemistry, University of Otago, Dunedin, 9054, New Zealand; University of Lethbridge, CANADA

## Abstract

HIV-1 utilises −1 programmed ribosomal frameshifting to translate structural and enzymatic domains in a defined proportion required for replication. A slippery sequence, U UUU UUA, and a stem-loop are well-defined RNA features modulating −1 frameshifting in HIV-1. The GGG glycine codon immediately following the slippery sequence (the ‘intercodon’) contributes structurally to the start of the stem-loop but has no defined role in current models of the frameshift mechanism, as slippage is inferred to occur before the intercodon has reached the ribosomal decoding site. This GGG codon is highly conserved in natural isolates of HIV. When the natural intercodon was replaced with a stop codon two different decoding molecules—eRF1 protein or a cognate suppressor tRNA—were able to access and decode the intercodon prior to −1 frameshifting. This implies significant slippage occurs when the intercodon is in the (perhaps distorted) ribosomal A site. We accommodate the influence of the intercodon in a model of frame maintenance versus frameshifting in HIV-1.

## INTRODUCTION

The ability of the ribosome to maintain reading frame fidelity during protein synthesis is fundamental. The tightly controlled mechanisms that maintain fidelity can, however, be superseded by programmed events, one of which is programmed ribosomal frameshifting (PRF) [1]. PRF involves tRNA slippage either 5´ (−1) or 3´ (+1) relative to the mRNA followed by continued translation in the new reading frame. PRF has mostly been studied in the context of eukaryotic viruses, and, more rarely, in bacteria, yeast and higher eukaryotes [2–8]. However, there is growing recognition of PRF as a regulatory mechanism used by both prokaryotes and eukaryotes ([9–11] and references therein). In the HIV-1 mRNA, −1 PRF results in translation of enzymatic domains and determines a specific ratio of enzymes to structural proteins critical for virus infectivity [12], [13].

PRF utilises a specific *cis*-element within the mRNA sequence where the change in reading frame occurs. In HIV-1 this sequence is a heptanucleotide string termed the ‘slippery sequence’ U UUU UUA (spaces separate codons in the 0 frame). Frameshift sites often contain additional RNA structural elements 3´ of the slippery sequence that further stimulate frameshifting; in most viruses the downstream structure is a pseudoknot, although stem-loops and protein binding sites can also be used [14–17]. For most strains of HIV-1, however, this structure is atypical and has been shown by NMR studies to be a stem-loop of two parts with an internal purine bulge, although recently it has been proposed that a network of pseudoknots exists in equilibrium with the widely accepted stem-loop structure [18–21]. While slippage can occur using a minimal 25-nucleotide sequence containing only a ‘slippery’ element [22], the downstream structure further stimulates the event to give frameshift efficiencies in the order of 5–10% dependent on the assay system used [23]. *In vivo*, the RNA helicase DDX17 modulates −1 PRF in HIV-1 [24]. The nucleotides immediately upstream of the slippery sequence, as well as distal elements of the HIV-1 RNA that affect the ribosomal density along the HIV-1 mRNA by modulating the rate of translation initiation, can also affect the efficiency of −1 PRF [25–28]. However, the frameshift efficiency measured *in vitro* with the element alone placed between different bicistronic reporter systems is surprisingly similar to the *in vivo* rate [29].

Despite intensive investigation, the molecular details of −1 PRF remain uncertain, with at least five plausible models proposed. Recently, kinetic studies have indicated that the ribosome may be induced into a conformation that disfavours translocation prior to −1 PRF [30], [31]. In most of these models, the heptanucleotide slippery sequence occupies the A and P sites of the ribosome as frameshifting occurs [15], [32].

We found previously that the codon immediately following the slippery sequence, that we have termed the intercodon, affects frameshifting mediated by just the slippery sequence in a simple bacterial system [33]. At that time, we proposed a post-translocational mechanism of tRNA slippage from the E and P sites because when the GGG intercodon was changed to a stop codon, frameshift efficiency decreased and was completely eliminated by up-regulating the specific prokaryotic release factor recognising only the cognate stop codon, RF2 [33]. This implied that the intercodon was present in the ribosomal A site prior to frameshifting. Interestingly, stop codons are found at the intercodon position immediately 3´ of slippery sequences in several backward frameshift elements, such as those of Rous sarcoma virus and barley yellow dwarf virus [2], [34], as well as at the positions of forward frameshifting in +1 PRF elements [4], [7].

We have undertaken an extended analysis of the role of the intercodon in the full-length HIV-1 frameshift element [35] placed between two different luciferase reporters in mammalian cultured cells, to better characterise its effect on frameshifting. We show that sense codon substitutions of the natural GGG intercodon significantly altered frameshift efficiencies. When this was substituted with a stop codon this efficiency was further modulated by over-expression of its decoding factors. Over-expression of the eukaryotic release factor, eRF1, decreased frameshift efficiency while cognate suppressor tRNA could increase frameshifting in competition with endogenous eRF1. Integrating these findings with current frameshift models, we propose a modified model of frameshifting in HIV-1 that takes into account the influence of the intercodon.

## MATERIALS AND METHODS

### Bioinformatics

HIV-1 sequences were downloaded from the Los Alamos National Laboratory (http://www.hiv.lanl.gov/) sequence database and aligned with MAFFT (v. 6.903b) [36]. Only sequences that encoded an intact, aligned slippery sequence (TTTTTTA) and that passed quality control checks used by the Los Alamos National Laboratory (i.e. were free of additional frameshift mutations, premature stop codons, and apparent hypermutation) were analysed. Sequences with ambiguous base calls in the intercodon were excluded. In total, 3534 of 4675 total sequences met these criteria and were used for further analysis. The BioPython (v. 1.59) toolset was used for further sequence analysis [37]. WebLogo (v. 2.8.2) was used to generate sequence logos [38].

### Reporter and expression vectors

The HIV-1 frameshift element variants containing the slippery sequence, intercodon and structural element of HIV-1 group M [35] were cloned into the pGL3s-hRLuc dual luciferase reporter vector [39], containing a 5´ human codon-optimised *RLuc* gene, the element, then a 3´ *Luc* ^*+*^ gene in the −1 frame. A control element for normalisation of data contained a nullified slippery sequence (CUUCUGA) with the 3´ *Luc* ^*+*^ gene in the original 0 frame such that output data reflect equimolar amounts of the two reporters synthesised. The human antizyme 1 (OAZ1) frameshift element of 120 nucleotides [7] was cloned into the dual luciferase vector but with the 3´ *Luc* ^*+*^ in the +1 frame to ensure Luc^+^ production only by a +1 frameshift. The same nullified construct (described above) was used for data normalisation. Constructs containing termination signals for readthrough assays were produced as described [39], with the 3´ *Luc* ^*+*^ gene in the original frame to ensure and measure continued translation only when termination failed. For data normalisation in this series, a construct containing the near-cognate UGG codon was used to produce equimolar amounts of both reporters. For the bicistronic fluorophore vector studies, the recoding sequences were excised and transferred into a vector between a 5´ EGFP reporter and 3´ DsRed.T4 reporter [40] in the appropriate frame [29].

eRF1 cDNA was cloned into the pcDNA3.1(+) vector (Invitrogen) using PCR primers (5´-ggaattcaagatggcggacgaccccagtgct-3´ and 5´-gctctagactagtagtcatcaaggtcaaa-3´) to create pcDNA-eRF1. The suppressor tRNA vectors, ptRNAoc (UAA), ptRNAam (UAG) and ptRNAop (UGA), and the wild-type ptRNAser (serine) were made as described and were a kind gift from Dr Olivier Jean-Jean (Université Pierre et Marie Curie, Paris, France) [41], [42]. Two siRNA target sequences [43], si90 and si1186 (modified from si1187), were cloned into the shRNA expression vector p*Silencer* 3.0-H1 (Ambion) according to the manufacturer’s instructions. A negative control (sh−ve), containing a sequence with limited homology to any portion of the human genome was provided by the manufacturer.

### Cell culture

COS-7 or HEK293T cells (obtained from the American Type Culture Collection) were maintained in DMEM (Invitrogen) containing 10% (v/v) fetal bovine serum (Invitrogen). For transfection of the dual luciferase vectors, cells were harvested at 80% confluence and seeded into 24-well plates at a density of 2 or 4 × 10^4^ cells/well (COS-7 and HEK293T respectively), and grown until 60% confluent. Vectors were transfected using the FuGENE 6 Transfection Reagent (Roche) at a ratio of 3 μl to 1 μg DNA. The reporter vector was transfected at 0.25 μg; and eRF1, shRNA and suppressor tRNA vectors up to 1 μg per well. For bicistronic fluorophore vectors, 1 × 10^5^ cells/well were seeded into 24-well plates and transfection of 0.5 μg reporter vector was performed at the same time as seeding (ratio FuGENE 6: DNA; 4 μl: 1 μg). Cells were incubated for 48 h after transfection of eRF1 and suppressor tRNA vectors, or 120 h after transfection of shRNA vectors with media changes at 60 h and 90 h, then washed twice with phosphate buffered saline and lysed with 100 μl Passive Lysis Buffer (Promega).

### Luciferase and fluorophore assays

Whole cell lysates were tested for *Renilla reniformis* luciferase (RLuc) activity using the described conditions [44] with modifications [45]. Firefly *Photinus pyralis* luciferase (Luc^+^) was assayed as described [46]. Lysate (10 μl) was mixed with RLuc or Luc^+^ assay buffer (50 μl) and relative light units were measured using an AutoLumat LB953 (EG&G Berthold). Relative frameshift efficiencies were calculated as: (Luc^+^_[test]_/RLuc_[test]_)/(Luc^+^_[control]_/RLuc_[control]_) × 100. The Student’s two-tailed *t*-test, assuming equal variance, was used to determine statistical significance, with an *a priori* α value of 0.05.

For the fluorophore assay, whole cell lysates were transferred to black 96-well plates (Greiner Bio-One) and relative fluorescence units were measured using a BMG POLARstar OPTIMA (BMG Labtech). EGFP (5´ reporter) and DsRed.T4 (3´ reporter) fluorescence was detected using 485 nm excitation/520 nm emission and 525 nm excitation/600 nm emission spectra, respectively. Relative frameshift efficiency was calculated as above.

### RNA isolation and qPCR

Total RNA from transfected cells was isolated using TRIzol^®^ (Invitrogen) according to the manufacturer’s instructions. Total RNA (10 μg) was incubated with 2 U DNase I (Roche) for 30 min at 37°C (25 mM Tris-HCl pH 7.2, 5 mM MgCl_2_, 0.1 mM EDTA), and DNase-treated and untreated RNA samples were analysed on formaldehyde agarose gels to determine quality and integrity. cDNA synthesis used total RNA (1 μg) with 250 ng random primers (Roche) and 200 U SuperScript™ III reverse transcriptase (Invitrogen). eRF1 and 18S rRNA transcript levels were determined using 2.5 ng of cDNA with a TaqMan^®^ Gene Expression Assay (eRF1 assay Hs01107358_g1, Applied Biosystems) or TaqMan Ribosomal Control Reagents (18S rRNA, Applied Biosystems). Reactions were performed using a 7500 Fast Real-Time PCR System (Applied Biosystems). TaqMan reactions were performed with ABsolute QPCR Low ROX Mix (Thermo Fisher Scientific). Expression levels were calculated using the comparative C_T_ method of relative quantification, after confirming that amplification efficiency of the target gene was within 5% of the reference gene efficiency.

### Western blotting and antibodies

Total cell lysates were harvested 48 h or 120 h after transfection as described. Total protein content was determined by BCA assay [47] and verified by Coomassie-stained polyacrylamide gels. Total protein (25 μg) was separated by 12.5% (w/v) SDS-PAGE and transferred to Protran nitrocellulose membranes (Whatman Schleicher & Schuell). Membranes were stained with Ponceau S staining solution (0.1% (w/v) Ponceau S, 5% (v/v) acetic acid) for 1–2 min, rinsed with dH_2_O, and the positions of lanes and marker bands were marked with a pencil. Membranes were then blocked for 1 h at 4°C in TBST (40 mM Tris-HCl pH 7.6, 150 mM NaCl, 0.05% (v/v) Tween 20) containing 5% (w/v) milk powder, incubated overnight at 4°C with rabbit anti-eRF1 [48] (1:10000 dilution, a kind gift from Dr Adam Geballe (Fred Hutchinson Cancer Research Center, Seattle, USA)) in TBST containing 5% (w/v) BSA, washed (3× with TBST), incubated (1 h at 4°C) with rabbit anti-β-actin (Sigma, 1:5000 dilution) and 2% (w/v) milk powder, and washed (3×, with TBST). Finally, the membranes were incubated (1 h at 4°C) with goat anti-rabbit–HRP (Sigma, 1:5000 dilution) in TBST containing 5% (w/v) milk powder, and washed (3×, with TBST) before ECL Western Blotting Detection Reagents (Amersham Biosciences) were applied according to the manufacturer’s instructions and the membranes exposed to X-ray film. eRF1 expression was quantified relative to β-actin and the non-transfected control using the ImageQuant™ TL software (Applied Biosystems).

## RESULTS

### The intercodon is highly conserved among viral isolates

Despite the error-prone nature of HIV replication, the frameshift element encompassing the slippery sequence and the stem-loop structural feature in the mRNA is highly conserved among HIV-1 sequences derived from patient blood samples [49], [50]. This observation is consistent with functional studies demonstrating that mutations perturbing −1 PRF dramatically effect replication efficiency of HIV-1. Moreover, recent observations indicate that only those mRNAs undergoing frameshifting are packaged into nascent HIV-1 virus particles, emphasising the importance of the PRF element [51], [52].

**Fig 1:**
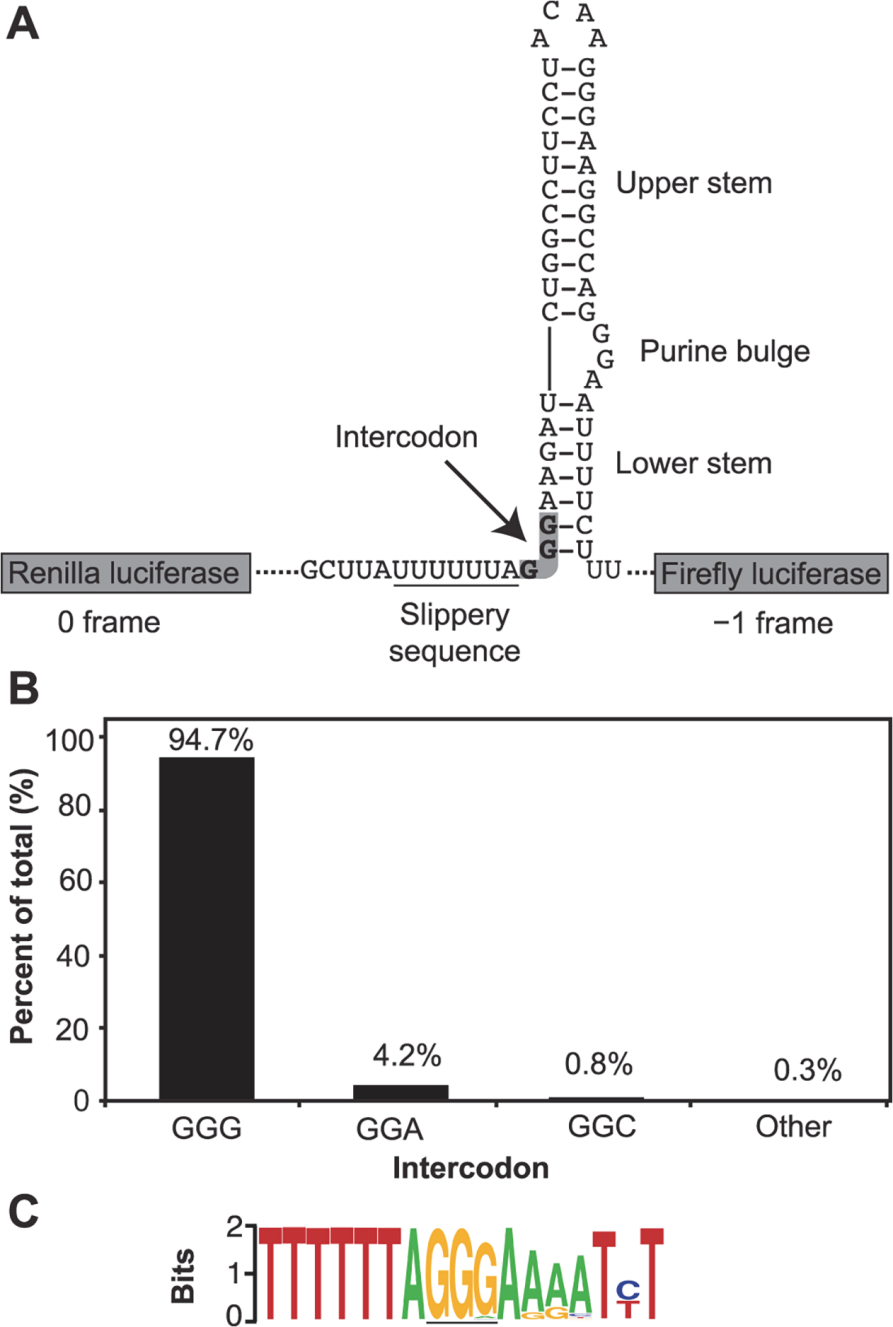
Context and conservation of the HIV-1 intercodon. A. The HIV-1 M-type frameshift element intercodon within the dual luciferase construct used in this study. B. Identity and frequency of the intercodon from sequences in the Los Alamos HIV sequence database as described in Methods. C. Sequence logo of the HIV-1 slippery sequence and downstream nucleotides spanning positions 2085–2101 (HBX2 numbering). The intercodon is underlined.

The conserved consensus sequence of the −1 PRF element of HIV-1 is shown in Fig. 1A. In an alignment of 3534 HIV-1 sequences, derived mostly from patient blood samples, we noted the intercodon following the slippery sequence was almost as highly conserved as the slippery sequence itself (Fig. 1B). Although minor variation was observed at the third position of the intercodon, 94.7% of sequences listed were GGG with G in this third position. The next most common codon was GGA (4.2%), and GGC was the only other codon with significant frequency (0.8%) (Fig. 1B). The remaining 0.3% may represent ‘noise’ from non-functional sequences not detected by the exclusion criteria. The consensus −1 PRF element derived from these data is shown in Fig. 1C.

In common with the nucleotides at or downstream of the −1 PRF slippage site, the intercodon encodes amino acids in two reading frames (GGG in the 0 frame and AGG in the −1 frame) and is at the very beginning of the extended stem-loop structure forming part of a structured RNA element. Our examination of the sequence downstream of the intercodon corresponding to the extended stem-loop showed it is not as highly conserved as the intercodon that forms the start of the stem-loop (Fig. 1C). The higher degree of conservation of the nucleotides of the intercodon compared to the other nucleotides of the downstream stem-loop led us to hypothesise that the intercodon might be directly involved in the translational frameshifting process.

### Does the intercodon play a role in frameshift efficiency?

In our earlier studies with bacterial ribosomes and the slippery sequence of the HIV-1 group M frameshift element [22], we replaced the natural GGG intercodon with a stop codon (as occurs for the intercodon in Rous sarcoma retroviral RNA) because at the time we had the capacity to express the test element in bacteria *in vivo* and over-express the decoding bacterial release factors (RF) [33]. The bacterial RF modulated frameshifting with codon specificity, implying that if slippage occurred during elongation it was when the intercodon was being decoded in the ribosomal A site. The −1 PRF constructs used in these studies however, lacked the downstream stem-loop and subsequent NMR structural studies of this secondary structural element suggested that the second and third nucleotides of the GGG intercodon are at the very start of a less stable lower region of an extended structure [18], [19].

**Fig 2:**
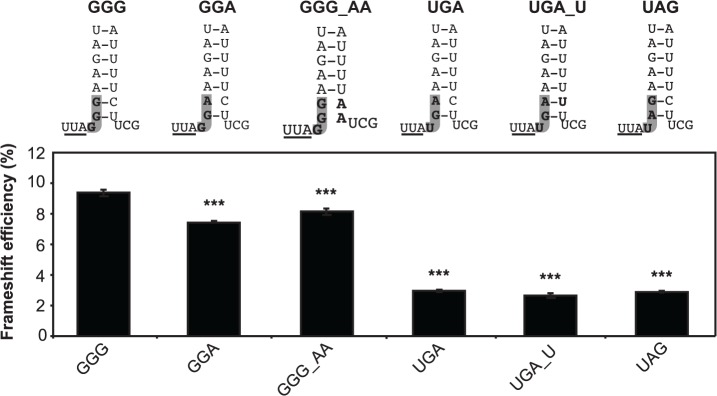
The identity of the intercodon influences −1 frameshift efficiency. Top: schematic representations of the base of the HIV-1 stem-loop, with the natural glycine codon and variants (left) and the substituted stop codon and variants showing the intercodon (grey) and any complementary stem-loop alterations (bold). The end of the slippery sequence is underlined. Below: frameshift efficiency of the natural intercodon and variants as assayed with the dual luciferase reporter system. GGG_AA and UGA_U indicate the intercodon (left part) and the modification to the complementary sequences in the stem-loop (right part). The mean ± standard error of the mean (SEM) for 18 replicates from three individual experiments in COS-7 cells is shown. ***P = < 0.001 compared to the GGG intercodon.

We first addressed in the current study whether the role of the GGG intercodon was solely structural in supporting frameshifting in the complete frameshift element [35] by analysing its role using a dual luciferase reporter system in cultured COS-7 cells (Fig. 2). With the natural GGG as the intercodon, the frameshift efficiency was 9.4%, consistent with the 5–10% range found for viral production *in vivo* [13], [49]. This suggested that, while there may be global structural effects on the −1 PRF efficiency involving other distant parts of the RNA, the element itself is the major determinant [28], [53].

Replacing GGG with GGA, the most common variant found in natural isolates, disrupts the lower stem structure and this change decreased frameshift efficiency to 7.8% (*P* < 0.001), indicating that disrupting the base of the stem weakens frameshifting to a minor degree. Efficiency was also somewhat reduced when the GGG codon was maintained but the lower stem was disrupted by substituting the natural UU with AA nucleotides on the opposing strand of the stem (GGG_AA).

Changing the intercodon to a UGA stop codon reduced apparent frameshift efficiency by ∼70% (*P* < 0.001), irrespective of whether the lower stem was disrupted (UGA), or restored by further substitutions in the opposing strand (UGA_U). Substituting a different stop codon (UAG for UGA) in the same position, which maintains the native structure of the lower stem, also reduced frameshifting by the same extent. Caution is warranted in interpreting these results, as the (non-frameshifted) short-form *Renilla* luciferase generated by this reporter, terminated at the intercodon, is truncated by 15 amino acids compared to the other constructs. The same relative results between these constructs were observed in a different bicistronic reporter system using the fluorophores EGFP and DsRed.T4 (S1 File), however [29]. Collectively, these data suggest that the GGG intercodon of the HIV-1 frameshift element influences frameshifting in at least two ways: in a minor fashion by promoting the lower stem structure and increasing the chance for −1 PRF to occur, but also more markedly by exerting another major influence that is not explained by current models of −1 PRF.

### Could base stacking explain frameshift modulation by the intercodon?

**Fig 3:**
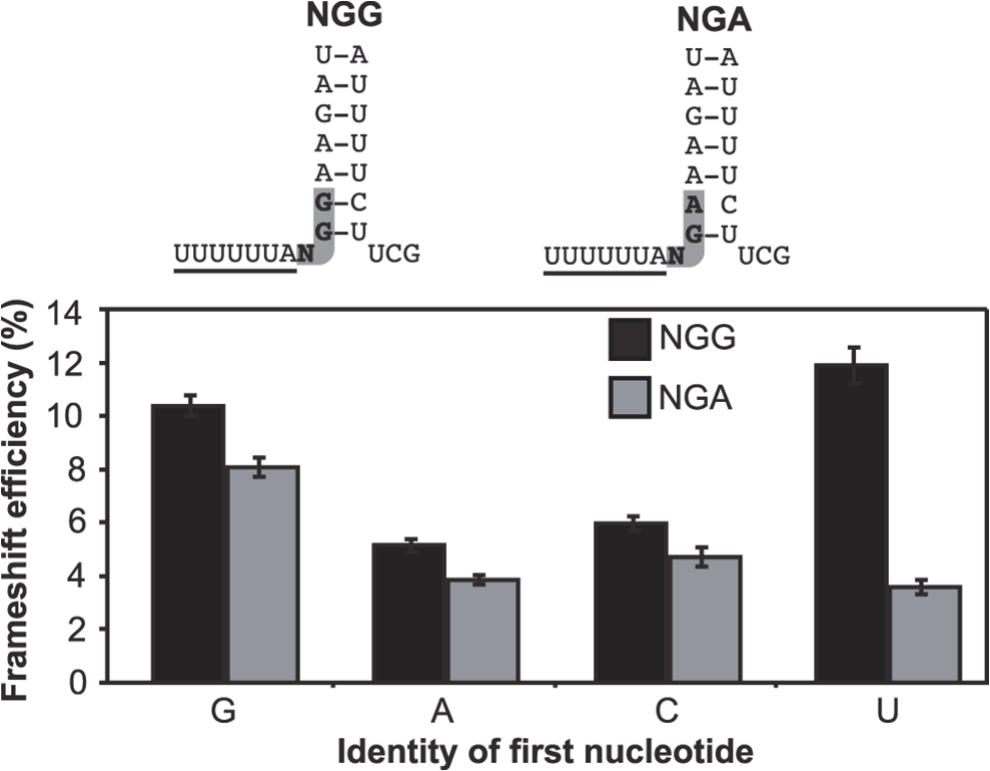
Frameshift efficiency is dependent on the identity of the first nucleotide of the intercodon. Frameshift efficiency for the NGG (black) and NGA (grey) contexts show the mean ± SEM for 33 replicates from five individual experiments in HEK293T cells.

The first base of the intercodon could affect frameshift efficiency by stacking on the A site codon (UUA) at the end of the slippery sequence as the ribosome stalls at the stem-loop. This model predicts that a purine would stack more easily than a pyrimidine due to its larger size and, therefore, would lower frameshift efficiency by providing greater stability for frame maintenance. By contrast, a pyrimidine in the same position would tend to be less effective [54]. Our results with GGG versus UGA (Fig. 2) were the opposite to that expected if such a stacking mechanism were dominant. We tested this further with a series of frameshift constructs with all four bases (A, G, C, T) for the first position of the intercodon, while the third base was either in the NGG or the NGA format. The order of frameshift efficiency for the NGG series was UGG>GGG>CGG>AGG whereas for the NGA series it was GGA>CGA>AGA>UGA (Fig. 3). In particular, the two purine bases, G and A, resulted in substantially different levels of frameshifting, which would not be predicted if base stacking were responsible for the major effect of the intercodon. Intriguingly, the two first base U codons, UGG (12% frameshifting) and UGA (3.5% frameshifting) were at the extremes. Notably, UGG is decoded by a tRNA while UGA is decoded by the eukaryotic protein release factor eRF1.

### Can the intercodon enter the decoding site prior to frameshifting?

We sought to test whether the intercodon occupied the decoding A site prior to frameshifting by using UGA as the intercodon and manipulating levels of the eukaryotic release factor, eRF1, similar to our previous experiments with bacterial ribosomes and prokaryotic release factors [33].

**Fig 4:**
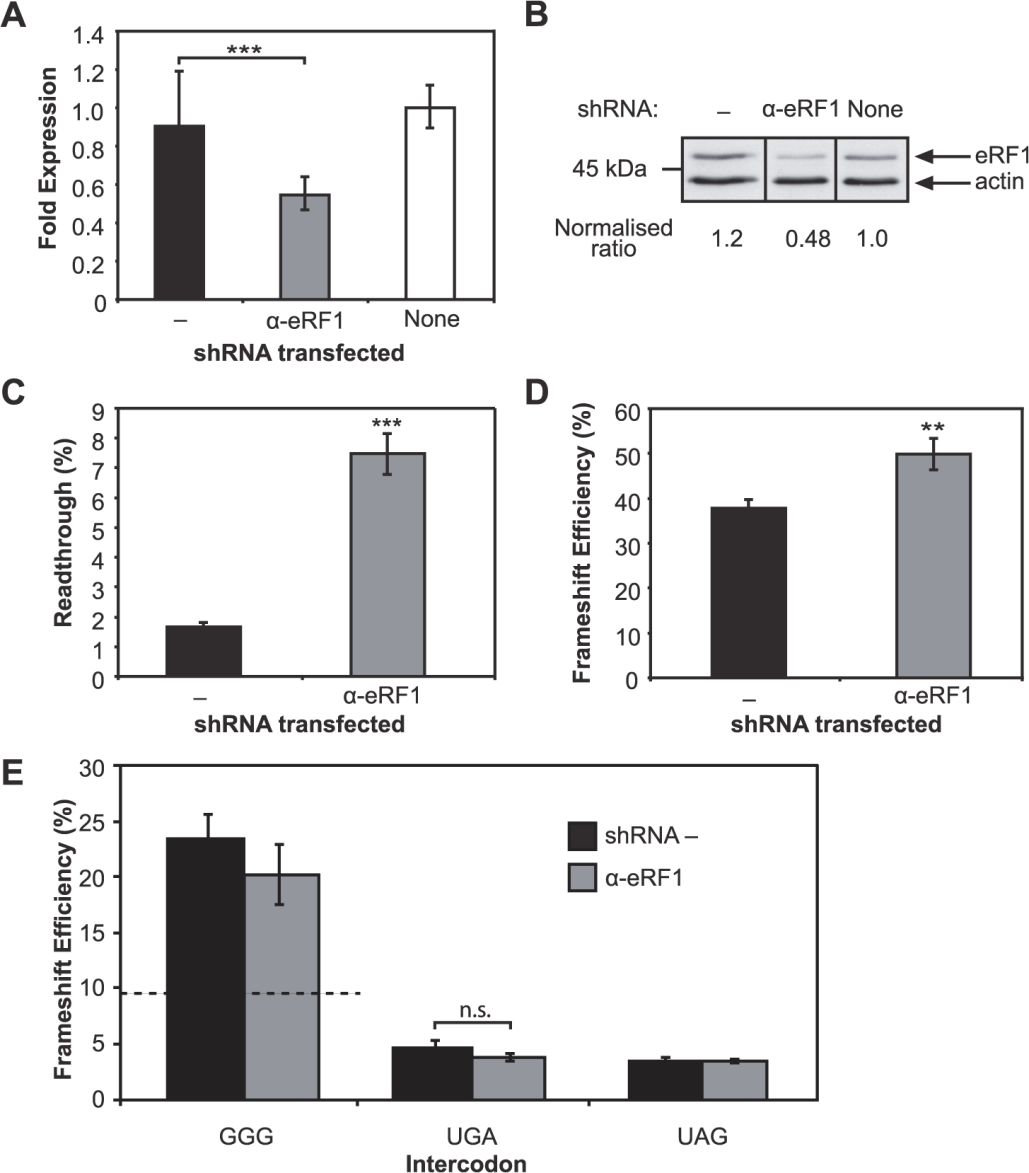
The effect of eRF1 depletion on termination and recoding. A. eRF1 transcript levels measured using quantitative real-time PCR. Results show eRF1 transcripts within RNA isolated from non-transfected HEK293T cells (‘none’), and eRF1 transcripts within RNA isolated from those transfected with α-eRF1 or negative control (−) vectors containing shRNAs. The mean ± standard deviation (SD) for six replicates is shown. B. Immunoblot of eRF1 in protein extracts from non-transfected HEK293T cells (‘none’), or cells transfected with α-eRF1 or negative control (−) vectors containing shRNAs. The ratios were calculated after normalisation to β-actin in each case and compared with the non-transfected control (1.0). Raw data is available in S1 Fig. C. Readthrough at a UGA test context (UGACAG). A dual luciferase construct with the two reporters in the same frame and the test stop signal separating them was co-transfected with the control and α-eRF1 shRNAs. Readthrough at the test stop signal was determined in each case (α-eRF1 and − control.) The mean ± SEM for 12 replicates from three individual experiments is shown. D. Effect of depletion of eRF1 on +1 frameshift efficiency at the human antizyme frameshift element. The mean ± SEM for eight replicates is shown. E. Effect of eRF1 depletion on frameshifting with the native GGG intercodon and substituted stop codon (UGA or UAG). The mean ± SEM for a minimum of 10 replicates from at least two independent experiments is shown. A dotted line indicates the level of −1 PRF in the absence of any shRNA (see Fig. 2). **P = < 0.01, ***P = < 0.001, n.s., not significant.

Initially, a strategy was used to evaluate the significance of eRF1 depletion on well-characterised translational systems. First, we tested whether down-regulating eRF1, using RNAi, would affect the natural competition that occurs between eRF1 and near-cognate tRNAs at stop codons *in vivo*. Dual luciferase constructs where the downstream luciferase sequence was in the same frame as the upstream luciferase reporter but separated by a stop signal were used for this purpose. We established that at the weakest stop signals, where the near-cognate tRNAs more effectively compete with the release factor, up to 3% readthrough can occur even with endogenous levels of eRF1. Cellular levels of eRF1 mRNA and protein were decreased two-fold 120 h after transfection of the vector expressing shRNAs (α-eRF1), compared with the shRNA negative control (−) (Fig. 4A & B). Cellular eRF1 protein was depleted using two vector-expressed shRNA sequences, si90 and si1186 (adapted from si1187) known to target eRF1 mRNA [43]. Both shRNAs reduced eRF1 mRNA and protein expression similarly (see S2 File for si1186 data). For both shRNAs, the physiological significance of reducing eRF1 was demonstrated with increased readthrough of ∼4.5-fold at the weak termination signal, UGACUG (*P* < 0.001) (Fig. 4C). This reduction in eRF1 resulted in a ∼30% increase in frameshift efficiency (*P* < 0.01) at the natural antizyme frameshift element where frameshifting naturally occurs with a UGA intercodon (Fig. 4D). This suggested that the cell was responding to the lowered concentration of eRF1 as expected.

Surprisingly, although no change was expected, the frameshift efficiency increased substantially with the naturally occurring HIV-1 PRF element (GGG intercodon) in response to shRNA expression independent of shRNA sequence. Frameshift efficiency increased 2–3 fold to 20–25% with both α-eRF1 and the shRNA negative control sequences (Fig. 4E). This was consistent with a previously observed general increase in −1 PRF upon dosage with small RNAs [55]. Non-physiological artefacts caused by shRNA expression could mask any specific effects of depleting eRF1 for the −1 PRF test system, in contrast to decoding competition at stop signals (readthrough and +1 PRF). Alternatively, a ∼2-fold reduction in the eRF1 protein observed may be insufficient to perturb the balance of forces promoting or opposing frameshifting. Indeed, the UGA and UAG intercodon constructs are not apparently affected by the shRNAs, with a typical frameshift efficiency of 4–5% recorded (Fig. 4E).

As the shRNA technology aimed at reducing eRF1 concentrations caused unexplained effects for the frameshifting assay, eRF1 was instead over-expressed in cells with an alternative technology. First, to establish that the endogenous eRF1 concentration was not saturating and that over-expressed eRF1 was active in the cultured cells, the stop codon readthrough reporter system was utilised as before. Subsequently, frameshifting at the antizyme +1 PRF element and the HIV −1 PRF element were tested.

**Fig 5:**
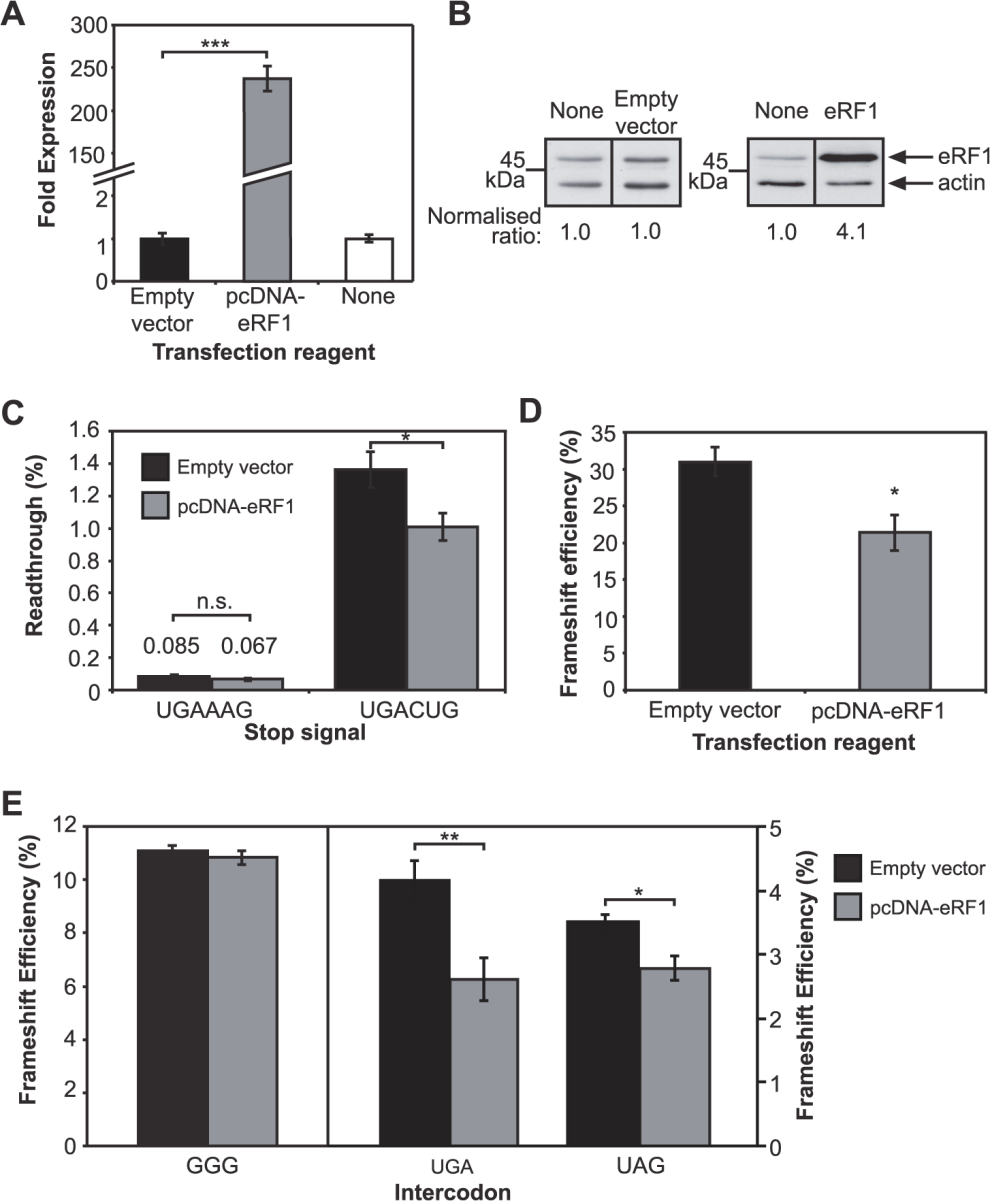
eRF1 over-expression influences frameshifting. A. eRF1 transcript levels measured using quantitative real-time PCR. Results show non-transfected HEK293T cells (‘none’), and those transfected with pcDNA-eRF1 vector or pcDNA3.1(+) with no insert (empty vector). The mean ± SD for six replicates is shown. B. Immunoblot of eRF1 expression in non-transfected HEK293T cells (‘none’), or cells transfected with pcDNA3.1(+) with no insert (empty vector) or pcDNA-eRF1 (eRF1). The ratios were calculated after normalisation to β-actin and comparison with the non-transfected control (1.0). Raw data are available in S2 and S3 Figs. C. Effect of over-expression of eRF1 on readthrough at UGA test contexts. The mean ± SEM for four replicates (UGAAAG) or 12 replicates from three individual experiments (UGACUG) is shown. Cells were transfected with either empty vector (black) or pcDNA-eRF1 (grey). Values are shown above the bars for the strong stop signal UGAAAG. D. Effect of over-expression of eRF1 on +1 frameshift efficiency at the human antizyme frameshift element. The mean ± SEM for six replicates is shown. E. Effect of over-expression of eRF1 on HIV-1 −1 frameshift efficiency with GGG intercodon and when it is substituted with a stop codon (UGA or UAG). The mean ± SEM for six replicates is shown. pcDNA (black), pcDNA-eRF1 (grey). *P = < 0.05, **P = < 0.01, ***P = < 0.001, n.s., not significant.

Cellular levels of eRF1 mRNA were increased 250-fold, resulting in a 4-fold increase in eRF1 protein 48 h after transfection (Fig. 5A and B). At a strong termination signal (**UGA**AAG) with very low readthrough (0.085%), the over-expressed eRF1 caused an apparent small decrease in readthrough that was not statistically significant (*P* > 0.05) (Fig. 5C). At a weak stop signal (**UGA**CUG) readthrough reduction was more robust, from 1.4% to 1.0% (*P* < 0.05). Co-transfection of the eRF1 vector, pcDNA-eRF1, with the antizyme +1 frameshift element resulted in a 30% lower +1 frameshift efficiency compared with control transfections (Fig. 5D). This demonstrated that over-expressing eRF1 can change the balance towards termination and against frameshifting at the UGA codon.

In contrast to a cell depleted of eRF1 with shRNA (see Fig. 4E), increasing the concentration of eRF1 had no effect on frameshift efficiency in the naturally occurring HIV-1 −1 PRF element that contains a non-cognate GGG intercodon (Fig. 5E, left). If the intercodon of the HIV-1 element was either of the cognate UGA or UAG stop codons, however, co-transfection with pcDNA-eRF1 resulted in a reduction of frameshift efficiency by 37% (*P <* 0.01) and 20% (*P <* 0.05), respectively (Fig. 5E, right). These data imply that the intercodon can be decoded prior to frameshifting, and is available for functional decoding by eRF1 when the GGG intercodon is replaced by a stop codon.

### Can tRNA-mediated decoding of the intercodon also occur prior to frameshifting?

In contrast to results presented in Fig. 5E, Kobayashi *et al*. (2010) reported that eRF1 is a general effector of −1 PRF frameshifting in HIV-1 when the intercodon is the natural GGG [55]. Although we did not observe this effect when eRF1 was over-expressed, given our uninterpretable results from depleting eRF1 we used suppressor tRNAs as an alternative decoding molecule of stop signals. Near-cognate tRNAs are naturally occurring potential suppressors of stop signals. They interact with termination codons by forming one or two base pairs with their anticodons but compete poorly with all but the weakest of stop signals. Artificially constructed cognate suppressor tRNAs that can form three Watson-Crick codon:anticodon interactions are highly competitive with eRF1 for decoding stop signals. If the changes in −1 PRF efficiency observed in Fig. 5E were mediated by a more general but unexplained property of eRF1, we would not expect to observe changes when using suppressor tRNAs. By contrast, if suppressor tRNAs modulated −1 PRF efficiency, then competition must be occurring between the suppressor tRNA and the endogenous eRF1 at the UGA intercodon within the ribosomal A site.

**Fig 6:**
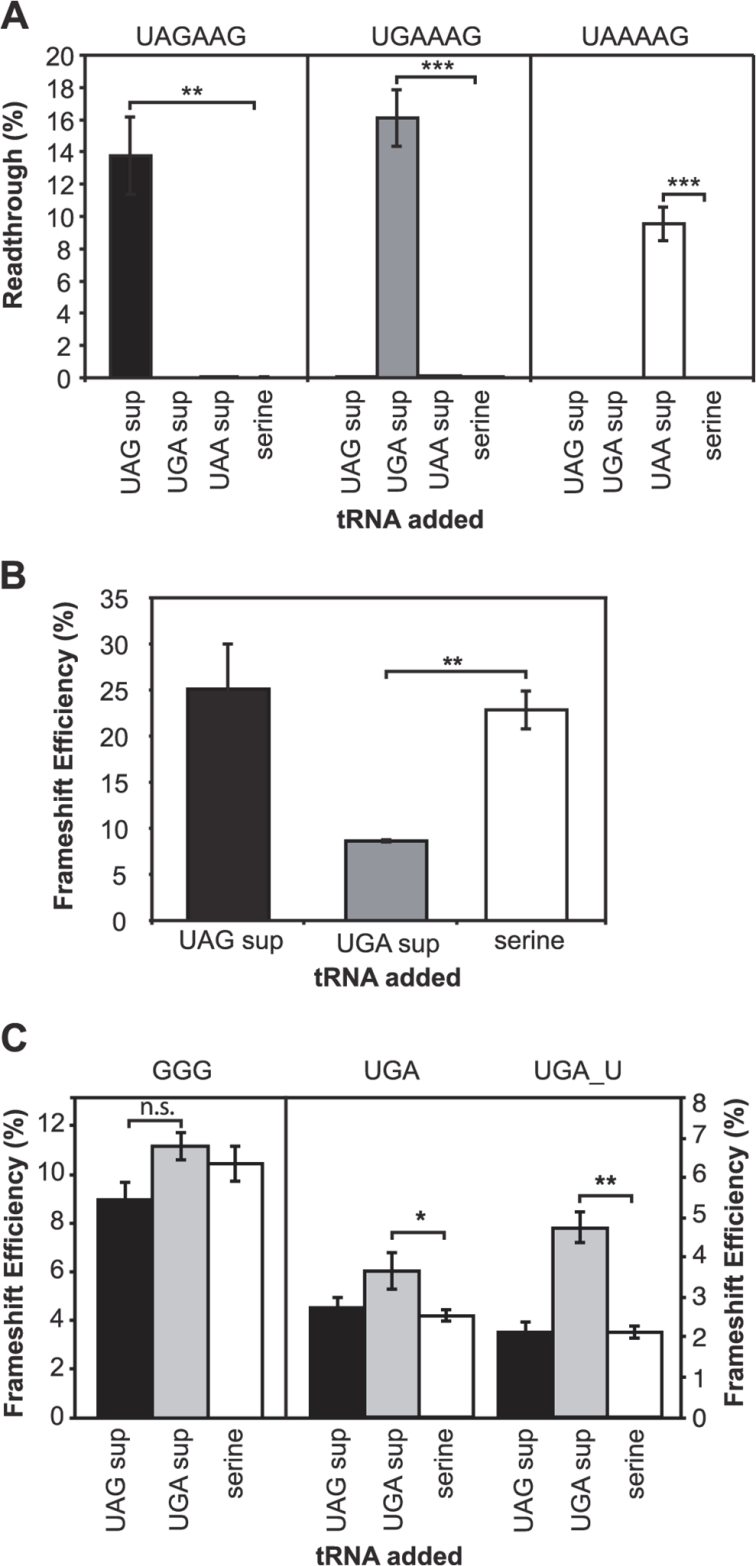
Specific intercodon suppressor tRNAs affect recoding efficiency. A. Readthrough at the test stop signals UAGAAG, UGAAAG and UAAAAG in HEK293T cells with cognate or non-cognate suppressor tRNAs (UAG [black], UGA [grey] or UAA [white]) and tRNA^Ser^ as a control (serine). The mean ± SEM for four replicates is shown. B. Effect of over-expressing cognate suppressor tRNA^UGA^, non-cognate tRNA^UAG^ or control tRNA^Ser^ on human antizyme +1 frameshift efficiency. The mean ± SEM for four replicates is shown. C. Effect on −1 frameshift efficiency of the HIV-1 element when UAG (black) or UGA (grey) suppressor tRNAs or the control tRNA^Ser^ (white) are expressed. Frameshift efficiency was measured for the cognate, non-cognate, and control tRNAs. Note the change in scales between when the intercodon is the native GGG (left) and UGA or UGA with a complementary mutation restoring the stem (UGA_U, right). The mean ± SEM for four replicates (GGG) or six replicates (UGA and UGA_U) is shown. *P = < 0.05, **P = < 0.01, ***P = < 0.001.

We utilised the human tRNA^Ser^ anticodon that had been previously modified to produce variants that would recognise UAA, UAG and UGA [41]. To first test their specificity in the cultured cells, readthrough of the efficient termination signals **UAA**AAG, **UAG**AAG and **UGA**AAG were measured in HEK293T cells with the cognate suppressor, the non-cognate suppressors and parent tRNA^Ser^ as the negative control. All test stop signals allowed only < 0.1% readthrough with co-expression of either the negative control tRNA^Ser^ or the non-cognate suppressors. Cognate suppressors increased readthrough to 9–16% at their respective termination signals 48 h after transfection (Fig. 6A).

The UGA cognate suppressor was competitive with eRF1 at the +1 antizyme frameshift site and reduced frameshift efficiency from 25% to 10% (*P* < 0.01), in contrast to the UAG non-cognate suppressor that produced no significant change (Fig. 6B). When either of the vectors encoding UAG or UGA suppressors or control tRNAs were co-expressed with the HIV-1 element containing the GGG codon, there were no statistically significant changes in −1 PRF efficiency (UAG suppressor) or no effect (UGA suppressor; Fig. 6C left). By contrast, with UGA or UAG as the intercodon, frameshift efficiency increased and with expected cognate specificity. Fig. 6C (right) shows an example with UGA and UAG suppressors and with the UGA PRF element but with ‘repair’ of the stem-loop (see Fig. 2) to correct the disruption at the bottom of the loop. These data reinforce the conclusion that the intercodon of the HIV-1 element is available in the vicinity of the A site for decoding by a tRNA or by a protein release factor before frameshifting occurs.

## DISCUSSION

### Role of the GGG intercodon in −1 frameshifting in HIV-1

We have used a reporter containing two luciferase-coding open reading frames, the second oriented in the −1 frame with respect to the first, and with the HIV-1 frameshift element placed between them, to study the function of the element in cultured cells. Specifically, the role of GGG, found immediately after the slippery sequence, has been evaluated for its role in frameshifting in HIV-1. This ‘intercodon’ is almost as highly conserved as the slippery sequence itself and is more conserved than the other nucleotides of the stem-loop (Fig. 1). The primary role of this intercodon in supporting −1 PRF efficiency does not appear to be either maintaining the structure of the lower stem-loop, or stacking of its first nucleotide on the last codon of the slippery sequence (Fig. 2 and Fig. 3). A strategy to test whether this intercodon has as its primary function an involvement in decoding before frameshifting utilised a stop codon substitution of the GGG intercodon. Both protein and tRNA decoding molecules for stop signals were then used to probe the decoding function of the intercodon. In this system, over-expression of the cognate decoding protein, eRF1, caused a reduction in—1 PRF (Fig. 5). This implied that the intercodon occupied the ribosomal A site and was available for decoding prior to at least a proportion of frameshift events. Suppressor tRNAs mitigated eRF1-induced reduction in −1 PRF efficiency, reinforcing the conclusion that the observed effects of eRF1 were specific to decoding activity in the A site of the ribosome.

By contrast to these clearly interpretable decoding experiments, the results from eRF1 knockdown by shRNAs were puzzling. First, levels of eRF1 were reduced with expected effects on readthrough at a test stop codon and +1 antizyme frameshifting with a specific shRNA targeting eRF1, but not with the control shRNA, as expected. However, translation of the HIV-1 frameshift element was affected differently from these other test systems. The control and test shRNAs both affected frameshifting with the native element (not containing a stop codon), and yet neither affected the element where GGG was substituted with UGA. We speculate that expression of shRNAs may have triggered the innate interferon response, activating the kinase PKR to decrease translation initiation efficiency by phosphorylation of eIF2α [56–58]. A slower translation rate increases frameshift efficiency in HIV-1 [28]. There is also evidence that the HIV-1 frameshift element is regulated by endogenous small RNAs [10]. Expressed shRNAs can compete with existing cellular small RNAs for the RNAi processing machinery [59].

Previously eRF1 knockdown has been shown to affect −1 PRF efficiency in HIV-1 with the GGG intercodon; that is, even when the element does not contain a stop codon [55]. We observed this as well, but also observed this in cells transfected with a control shRNA sequence, indicating a non-specific effect in our system. While it is difficult to reconcile fully the two data sets, we note that the former study had a greater eRF1 knockdown (∼80%) compared with our more modest 50%. The HIV-1 frameshift element and reporter translations may have some unexplained interaction with the shRNAs not seen with the readthrough or +1 antizyme translations (discussed above).

### The translation product resulting from frameshifting in HIV-1

Amino acid sequencing data have shown that the protein product of −1 frameshifting from the HIV-1 PRF element is heterogeneous, indicating the presence of multiple pathways leading to −1 PRF in HIV-1. The amino acid corresponding to the last codon of the slippery sequence (UUU, coding for phenylalanine in the −1 frame; or UUA, coding for leucine in the 0 frame) has been found to be 20–30% phenylalanine and 70–80% leucine in the protein product of −1 PRF [3], [60], [61]. Incorporation of phenylalanine (the minority product) in this position would occur before the intercodon reaches the A site if the ribosome frameshifts prior to peptidyl transfer, resulting in UUU occupying the A site and being decoded by tRNA^Phe^. This pathway has been described in detail by Liao *et al*. (2011) [60]. Incorporation of leucine, by contrast, could occur either before or while the intercodon occupies the A site [32], [60]. A possible pathway for the latter scenario, in which leucine is incorporated into the peptide chain while the intercodon occupies the ribosomal A site, is described below.

We observed a 4-fold decrease in −1 PRF efficiency from 10% with a GGG intercodon to 2.5% with the substituted UGA intercodon when eRF1 was over-expressed. This implies that a significant number of the HIV-1 frameshifting events could be occurring when the intercodon is in the ribosomal decoding (A) site. This is in good agreement with the semi-quantitative amino acid sequencing data discussed above [3], [60], [61].

### A modified model of frameshifting in HIV-1

**Fig 7:**
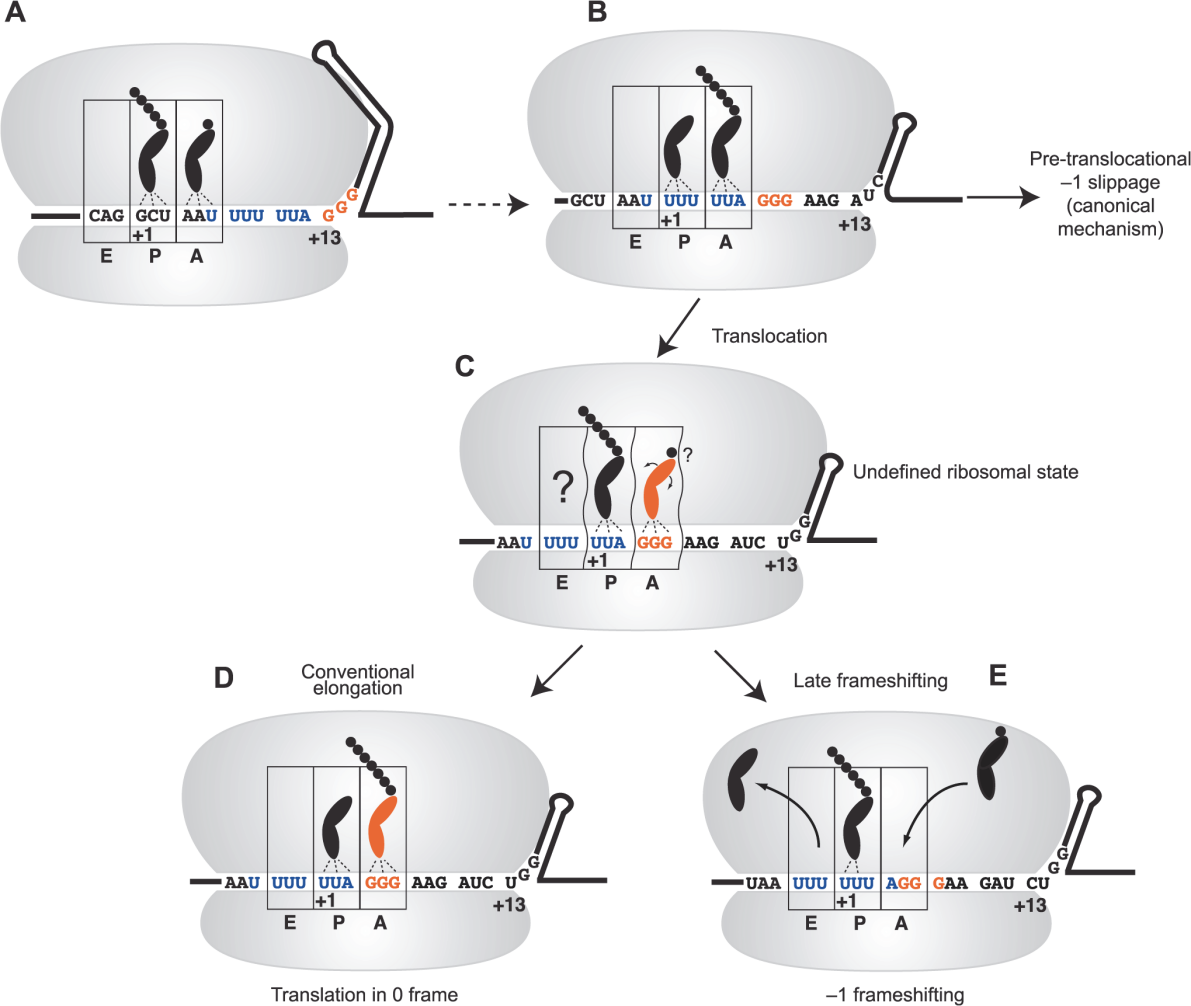
A modified model for −1 frameshifting in HIV-1. A. The first nucleotide (U) of the slippery sequence (blue), as part of the ‘BCX’ codon [26], is positioned in the A site. The extended stem-loop is at the entrance to the ribosome, with the first nucleotide of the intercodon (orange) in the +13 position. B. The lower stem of the structure unwinds and allows the slippery sequence into the decoding centre. The stable upper stem-loop is positioned near the entry channel of the ribosome. Tension arising from resistance to unwinding may allow slippage from the A and P sites at this stage. C. After partial unwinding of the stable upper stem-loop, binding of tRNA^Gly^ to the intercodon, perhaps in a distorted state, competes with −1 frameshifting. D. The tRNA^Gly^ bound to the intercodon is decoded and translation proceeds in the 0 frame. This is the most common event, resulting in the translation of the Gag product. E. If the tRNA^Gly^ is not decoded, −1 frameshifting by the E and P site tRNAs occurs to relieve tension on the mRNA, resulting in the translation of Gag-Pol. An incoming tRNA^Arg^ is shown.

We propose a modified model of HIV-1 frameshifting to accommodate a role for the intercodon in slippage. This is nonetheless consistent with existing models of −1 PRF, where tension created by opposing forces of forward translocation and resistance to unwinding of the stem-loop is a crucial determinant of frameshift efficiency (Fig. 7) [62–64]. mRNA enters the ribosome 13±2 nucleotides from the first (+1) nucleotide of the P site codon [63], [65], [66]. In this scenario, the GGG intercodon would be positioned at the entrance to the mRNA channel when the first nucleotide of the slippery sequence has just entered the A site (Fig. 7A). Indeed, the distance between the slippery sequence and the structural element has been implicated as contributing to frameshifting [23], [64].

For translation to proceed through the secondary structural stem-loop in HIV-1, the thermodynamically weaker lower stem would unwind to allow the slippery sequence to occupy the P and A sites (UUU UUA) (Fig. 7B). Slippage from the P and A sites may now occur as previously observed [26], [67]; that is, a portion of ribosomes undergo canonical pre-translocational frameshifting. The data presented in this paper indicates, however, that in a substantial fraction of ribosomal passages the translating ribosome would maintain the existing reading frame and would simply be ‘slowed down’ by the building tension (Fig. 7C) [64].

As the intercodon occupies the A site, the stable upper stem blocks the mRNA entry tunnel and resistance to the forward movement of the RNA would further slow the progress of the ribosome. In our model, this resistance created within the HIV-1 element would disrupt the canonical elongation events of some of these ribosomes when the intercodon occupies the A site (Fig. 7C). As already suggested [26], an incomplete translocation of the tRNAs and mRNA may occur resulting in distortion of the tRNAs in the P and E sites, similar to that seen with the P site tRNA in cryo-EM structures [63], and there may be a ‘disturbed’ occupation of the A site by the intercodon (Fig. 7C) [30].

It is difficult to predict what the ribosome complex might look like in these atypical states created by the unusual elements within the RNA at frameshift sites. For example, it has been reported recently that a prokaryotic −1 PRF-stimulating element, when positioned at the entrance channel of the *E*. *coli* ribosome, can trigger an unusual ‘hyper-rotated’ state [68]. The tRNA^Gly^ interacting with the GGG intercodon may be distorted in the A site, essentially unstable and poorly accommodated for peptide bond formation [32]. Consistent with this model, ribosomal mutations that increase the rate of accommodation negatively affect −1 PRF efficiency [67].

A second round of frameshifting, occurring as the intercodon occupies the A site and the stable upper stem-loop resides at the beginning of the mRNA entry tunnel, then would be facilitated by a partially distorted intercodon and may be thought of as a competition between conventional decoding and frameshifting (Fig. 7D and Fig. 7E). Different tRNAs, with their unique primary structures, modifications, and cellular concentrations would be predicted to have different binding capabilities to a distorted A site codon (as in Fig. 7C). Consistent with this prediction, we observed a variation in frameshift efficiency among different intercodons decoded by different tRNAs (Fig. 3). UGG (tRNA^Trp^) was less capable, and AGG and CGG (tRNA^Arg^) more capable of maintaining the reading frame compared with GGG (tRNA^Gly^). A third base A (GGA, AGA and CGA intercodons) all produced significantly lower levels of frameshifting (i.e. better frame maintenance) than their third-base G counterparts, perhaps because A•U wobble-base pairing is stronger than G•U. Alternatively, the ribosome may undergo an incomplete translocation at this stage, resulting in elongation continuing in the −1 reading frame [31]. In a +1-frameshifting system, out-of-frame tRNAs are capable of influencing frameshifting, indicating substantial plasticity of the ribosome in these unusual circumstances [69].

A special circumstance is created when the intercodon is a stop codon at +1 or −1 frameshift sites, as occurs naturally in bacterial RF2 (+1 PRF), human antizyme (+1 PRF), Rous sarcoma virus (−1 PRF), and barley yellow dwarf virus (−1 PRF). For our studies a stop codon as the intercodon was artificially created for the HIV-1 site. In these cases, the decoding molecule is not a tRNA but a protein release factor, which, unlike a tRNA, does not interact solely with the triplet codon but also the downstream sequence of the mRNA [70], [71]. Of interest is that the release factor has been shown to recognise disturbed stop codons in the A site of the bacterial ribosome [72]. Such an extended interaction between the release factor and mRNA:ribosome complex would be expected to compete strongly with frameshifting if the stop codon was occupying the ribosomal A site—more so than tRNA with the canonical mRNA:tRNA codon:anticodon interaction (and particularly if the intercodon is distorted in the A site). Consistent with this prediction, we found that the UGA suppressor tRNA was less able to prevent frameshifting than eRF1 at the HIV-1 site (Fig. 6C). This effect would be predicted to depend upon the efficiency of decoding by the molecule during accommodation, after occupying the ribosomal decoding site, in addition to the concentration of the decoding molecule in the cell. In a distorted decoding site it is not easy to predict which decoding molecule (eRF1 or suppressor tRNA) would be the more efficient decoding molecule.

By contrast, at the natural antizyme intercodon, where there is a weak stop signal (UGAC) eRF1 was a poorer competitor than a UGA suppressor tRNA so frameshifting is high (Fig. 6B). In terms of an ability to maintain the original frame at the HIV-1 site, the UGA suppressor tRNA fell between relatively inefficient competitors of frameshifting such as the natural tRNA^Gly^ (GGG) and tRNA^Trp^ (UGG), and stronger competitor tRNA^Arg^ isoacceptors (for AGG/AGA and CGG/CGA).

Alternatively, the measured difference in frameshift efficiency observed in our system could be influenced by a difference in activity of the short form of the *Renilla* luciferase enzyme, terminated at either the intercodon (if changed to a stop codon) or the downstream 0-frame stop codon. When utilising a suppressor tRNA to compete with endogenous eRF1, a minor fraction of the non-frameshifted product would be expected to bypass the 0-frame stop codon and terminate at a downstream stop signal by readthrough; a difference in activity or stability between these two forms of the short enzyme could in principal account for the apparent competition effect observed in Fig. 6C and this possibility cannot be discounted. As only a fraction of the short enzyme species would have this short extension, however, a substantial change in specific activity would be required to yield the statistically significant difference seen in Fig. 6C.

For the mRNA to disengage from its two tRNAs there must be significant perturbations to the typical precise ribosomal conformations at each step of the elongation cycle. Despite this, in 90–95% of cases the decoding molecule tRNA^Gly^ can successfully bind and decode the GGG intercodon, allowing the ribosome to escape frameshifting by overcoming resistance from the unwinding of the upper stem and continue to translate the mRNA in the original frame (Fig. 7D). However, in those ribosomes that do not ‘escape’ when the intercodon is in the A site, the tension placed on the mRNA is instead relieved by disruption of the codon:anticodon interactions of the P (and possibly E site) tRNA(s) to result in a ‘spring-like’ movement of the tRNAs and re-pairing in the −1 frame [62].

The importance of the E site for frame maintenance has been established with bacterial ribosomes by analysis of the bacterial RF2 +1 frameshift mechanism that relies on destabilisation of E site tRNA binding [73], and from the fact that weaker E site tRNA interactions (A•U rather than G•C) correlate with higher +1 PRF in *E*. *coli* [74]. Indeed, the E site plays a general role in frame maintenance in prokaryotic ribosomes [75]. Three 16S rRNA mutations located in helices 21 and 22 increase −1 PRF with the HIV-1 element [26], and these are involved in E site tRNA binding. The model presented in Fig. 7 is consistent with these observations, although our experimental data are not directly informative in this regard. For HIV-1, slippage might occur first at the E site followed by slippage at the P site [26], [76], as opposed to the simultaneous-slippage model proposed originally [3]. Additionally, in yeast Bekaert and Roussel (2005) showed bioinformatically when X XXY YYZ is in the P and A ribosomal sites the two nucleotides immediately upstream in the E site are highly biased, suggesting an extended signal. In yeast, high frameshift efficiency corresponded to a pseudouridine at tRNA position 39 that might decrease stability of the P or E site tRNA interactions [77].

### Heterogeneity among −1 programmed ribosomal frameshift elements

HIV-1 is unusual among frameshifting retroviruses in that it lacks a pseudoknot stimulator of PRF. Early work on the Rous sarcoma virus −1 PRF element, which contains a pseudoknot, found that mutations in the intercodon (naturally a stop codon in this instance) did not affect frameshift efficiency. Amino acid sequencing by Edman degradation did not detect the predicted product from ‘late’ −1 PRF that could be translated if the intercodon were occupying the A site in the Rous sarcoma virus frameshift element [78], [79]. We propose that the late −1 PRF event we have observed in the M-type HIV-1 element may not be common to other frameshifting retroviruses that utilise a pseudoknot structural element, a hypothesis that we are currently investigating.

Using kinetic and single-molecule fluorescence data, three recent studies, utilising *in vitro* translation systems, have observed frameshifting occurring during translocation in the prokaryotic *dnaX* and infectious bronchitis virus elements on bacterial ribosomes [30–32]. While the elements examined here had modifications to give a comparatively high frameshift efficiency (> 70%), our proposed model of ‘late’ lower efficiency of 5–10% frameshifting in HIV shares some similarities with the proposed mechanism of −1 PRF in *dnaX*, in which the A site codon can be ‘sampled’ by tRNA with the ribosome in an abnormal rotated state [32].

Several reasons suggest caution, however, in direct comparisons of our data with data from these studies. The *dnaX* and infectious bronchitis virus elements both contain a two-codon spacer between the slippery sequence and the structured RNA element, while the HIV-1 M-type element contains only a one-base spacer, or alternatively an 8-base spacer if only the more stable upper stem-loop is considered. This may lead to a pause in the case of the elements with large spacers, compared with a decrease in translation rate occurring earlier at the HIV-1 element until a more marked pause occurs when the stable upper stem-loop abuts the ribosome [63]. The ribosome has two mechanisms of helicase activity, which may differ with respect to these two downstream structures [65].

Recently, eukaryotic-specific contacts between the ribosome and mRNA in the entry channel, as well as a different eukaryotic-specific subunit rearrangement during decoding, have been reported [80]. By contrast, prokaryotic elements contain an upstream internal Shine–Dalgarno sequence which helps pause the ribosome. There is known allosteric communication between the A site and the Shine–Dalgarno sequence, which may contribute to differences in the mechanism of −1 PRF in these cases [81]. These differences underscore the diversity among −1 PRF elements found in nature, exemplified by the differences in the HIV-1 and studied prokaryotic −1 PRF elements.

### Conclusion

During HIV-1 evolution the GGG intercodon may have been selected to give a level of frameshifting that produces a ratio of enzyme to structural proteins that best supports virus replication. The work presented in this study supports the hypothesis that −1 PRF in HIV-1 occurs relatively frequently with the slippery sequence at the E and P sites, and highlights that events during decoding of the intercodon are critical for frameshift efficiency. Namely, competition between decoding the GGG intercodon (resulting in regular translation with a maintained reading frame) and −1 PRF occurs in a portion of HIV-1 frameshift events. This scenario can be integrated into existing models of frameshifting and supports the notion that frameshifting in HIV-1 is a heterogeneous process.

## Supporting Information

S1 Fig Raw data relating to Fig. 4B. From left to right in Fig. 4B, bands from lanes ‘5d 1.0 μg si-ve’, ‘5d 1.0 μg si90’, and ‘5d 0 μg’ are shown. The leftmost lane contains markers corresponding proteins of, from top to bottom, 116.2 kDa, 97.4 kDa, 66.2 kDa, 45 kDa, 31 kDa, 21.5 kDa, 14.4 kDa, and 6.5 kDa. The 21.5 kDa molecular weight standard was marked as a doublet band in this transfer.
(TIF)

S2 Fig Raw data relating to Fig. 5B, leftmost two lanes. Bands from lanes ‘0’ and ‘1.0’ are shown in Fig. 5B as the leftmost ‘None’ and ‘Empty vector’ boxes, respectively. The leftmost lane contains markers corresponding to molecular weight standards of, from top to bottom, 116.2 kDa, 97.4 kDa, 66.2 kDa, 45 kDa, 31 kDa, 21.5 kDa, and 14.4 kDa. The last marker likely represents the bromophenol blue dye front.
(TIF)

S3 Fig Raw data relating to Fig. 5B, rightmost two lanes. Bands from lanes ‘0’ and ‘1.0’ are shown in Fig. 5B as the rightmost ‘None’ and ‘eRF1’ boxes, respectively. Markers are the same as for S2 Fig.
(TIF)

S1 File Measuring −1 PRF elements in a bifluorescent reporter system. The reporter system contains two fluorescent proteins, separated by the HIV-1 group M −1 PRF element, with the downstream protein positioned in the −1 frame [29]. ‘Null0’ refers to an in-frame control reporter, and ‘controls’ refers to background fluorescence values from mock-transfected cell lysate. The mean ± SEM frameshift efficiencies for three replicates from one experiment are shown in the included graph.
(XLSX)

S2 File Raw data relating to Fig. 4 and alternative shRNA. A. Quantitative PCR. Threshold cycle (Ct) values for target gene (TG) eRF1 and reference gene (RG) 18S rRNA. Two pooled replicates were tested, each containing cell lysate from four individually transfected wells (a total of eight transfections). Three technical replicates were made for each pooled sample. Data were analysed using the comparative CT method to give fold differences and standard deviations. B, baseline; T, threshold; NTC, no template control; 0ug, non-transfected control. ‘-ve’, ‘90’ and ‘1186’ correspond to the shRNA vectors that were transfected. C. Stop codon readthrough. Values for *Renilla* luciferase (Rluc) and firefly luciferase (Luc+) are in relative light units. Background (bkgd) values (cell lysate alone) were subtracted from each measurement. The percentage of readthrough of the stop signal for each sample was calculated using the corresponding sense readthrough control (RT) from each replicate plate. Replicates are from three individual experiments. ‘-ve’, ‘90’ and ‘1186’ correspond to the shRNA vectors that were transfected. D. Antizyme frameshifting. Values for *Renilla* luciferase (Rluc) and firefly luciferase (Luc+) are in relative light units. Background (bkgd) values (cell lysate alone) were subtracted from each measurement. The relative frameshift efficiency for each sample was calculated using the corresponding average readthrough control from each replicate plate. Replicates are from one experiment. The value in bold italics was not included as it was greater than two standard deviations above the mean. Az, antizyme; RT, readthrough control. ‘-ve’, ‘90’ and ‘1186’ correspond to the shRNA vectors that were transfected. E. HIV frameshifting. Values for *Renilla* luciferase (Rluc) and firefly luciferase (Luc+) are in relative light units. Background (bkgd) values (cell lysate alone) were subtracted from each measurement. The relative frameshift efficiency for each sample was calculated using the corresponding readthrough control from each replicate plate. Replicates are numbered according to individual experiments (replicates 1–4, 5–8, 9–12 and 13–18). ‘-ve’, ’90’ and ‘1186’ correspond to the shRNA vectors that were transfected.
(XLSX)

S3 File Raw data relating to Fig. 1. (DOCX)

S4 File Raw data relating to Fig. 2. HIV frameshifting. Values are relative frameshift efficiency for each sample. Replicates are from three individual experiments. For UGA_U data: Values for *Renilla* luciferase (Rluc) and firefly luciferase (Luc+) are in relative light units. Background (bkgd) values (cell lysate alone) were subtracted from each measurement. The relative frameshift efficiency for each sample was calculated using the average readthrough control. Replicates are from one experiment.
(XLSX)

S5 File Raw data relating to Fig. 3. HIV frameshifting. Values for *Renilla* luciferase (Rluc) and firefly luciferase (Luc+) are in relative light units. Background (bkgd) values (cell lysate alone) were subtracted from each measurement. The relative frameshift efficiency for each sample was calculated using the corresponding average readthrough control from each replicate plate. Replicates are numbered according to individual experiments (replicates 1–4, 5–12, 13–18, 19–33 and 19–36 (RT), 34–41 and 37–40 (RT)).
(XLSX)

S6 File Raw data relating to Fig. 5. A. Quantitative PCR. Samples were from transfection of increasing amounts of pcDNA or pcDNA-eRF1 vector (0 μg to 1.5 μg). Threshold cycle (Ct) values for target gene (TG) eRF1 and reference gene (RG) 18S rRNA. Two pooled replicates were tested, each containing cell lysate from four individually transfected wells (a total of eight transfections). Three technical replicates were made for each pooled sample. Data were analysed using the comparative CT method to give fold differences and standard deviations. B, baseline; T, threshold; NTC, no template control. C. Stop codon readthrough. Values for *Renilla* luciferase (Rluc) and firefly luciferase (Luc+) are in relative light units. Background (bkgd) values (cell lysate alone) were subtracted from each measurement. The percentage of readthrough for each sample was calculated using the corresponding readthrough control from each replicate plate. Replicates are from one experiment for UGAAAG samples, and three individual experiments for UGACUG samples. p, pcDNA 3.1(+) with no insert (empty vector); e, pcDNA-eRF1. D. Antizyme frameshifting. Values for *Renilla* luciferase (Rluc) and firefly luciferase (Luc+) are in relative light units. Background (bkgd) values (cell lysate alone) were subtracted from each measurement. The relative frameshift efficiency for each sample was calculated using the corresponding average readthrough control (RT) from each replicate plate. Replicates are from one experiment. Value in bold italics was omitted as it was greater than two standard deviations below the mean. Az, antizyme; p, pcDNA 3.1(+) with no insert (empty vector); e, pcDNA-eRF1. E: HIV frameshifting. Values for *Renilla* luciferase (Rluc) and firefly luciferase (Luc+) are in relative light units. Background (bkgd) values (cell lysate alone) were subtracted from each measurement. The relative frameshift efficiency for each sample was calculated using the corresponding readthrough control (RT) from each replicate plate. Replicates are from one experiment. Values in bold italics were not included as they were greater than two standard deviations above or below the mean. Two individual clones, labelled 1 and 2, were used for the UAG reporters. p, pcDNA 3.1(+) with no insert (empty vector); e, pcDNA-eRF1.
(XLSX)

S7 File Raw data relating to Fig. 6. A: Stop codon readthrough. Values for *Renilla* luciferase (Rluc) and firefly luciferase (Luc+) are in relative light units. Background (bkgd) values (cell lysate alone) were subtracted from each measurement. The percentage of readthrough for each sample was calculated using the corresponding average readthrough control from each replicate plate. Replicates are from one experiment. Values in bold italics were not included as they were greater than two standard deviations above or below the mean. CAG was the UAA readthrough control, UAU the UAG readthrough control, and UGG the UGA readthrough control. ser, serine tRNA; oc, ochre (UAA suppressor tRNA); am, amber (UAG suppressor tRNA); op, opal (UGA suppressor tRNA). B: Antizyme frameshifting. Values for *Renilla* luciferase (Rluc) and firefly luciferase (Luc+) are in relative light units. Background (bkgd) values (cell lysate alone) were subtracted from each measurement. The relative frameshift efficiency for each sample was calculated using the corresponding average readthrough control (RT) from each replicate plate. Replicates are from one experiment. The value in bold italics was not included as it was greater than two standard deviations above the mean. Az, antizyme; ser, serine tRNA; am, amber (UAG suppressor tRNA); op, opal (UGA suppressor tRNA). C: HIV frameshifting. Values for *Renilla* luciferase (Rluc) and firefly luciferase (Luc+) are in relative light units. Background (bkgd) values (cell lysate alone) were subtracted from each measurement. The relative frameshift efficiency for each sample was calculated using the corresponding average readthrough control (RT) from each replicate plate. Replicates are from one experiment. The value in bold italics was not included as it was greater than two standard deviations above the mean. ser, serine tRNA; am, amber (UAG suppressor tRNA); op, opal (UGA suppressor tRNA).
(XLSX)
