# Supplementary figures and images for: The Highly Conserved Codon following the Slippery Sequence Supports −1 Frameshift Efficiency at the HIV-1 Frameshift Site
Source: PLoS One. 2015 Mar 25;10(3):e0122176. doi: 10.1371/journal.pone.0122176 (PMC4373837; doi:10.1371/journal.pone.0122176)

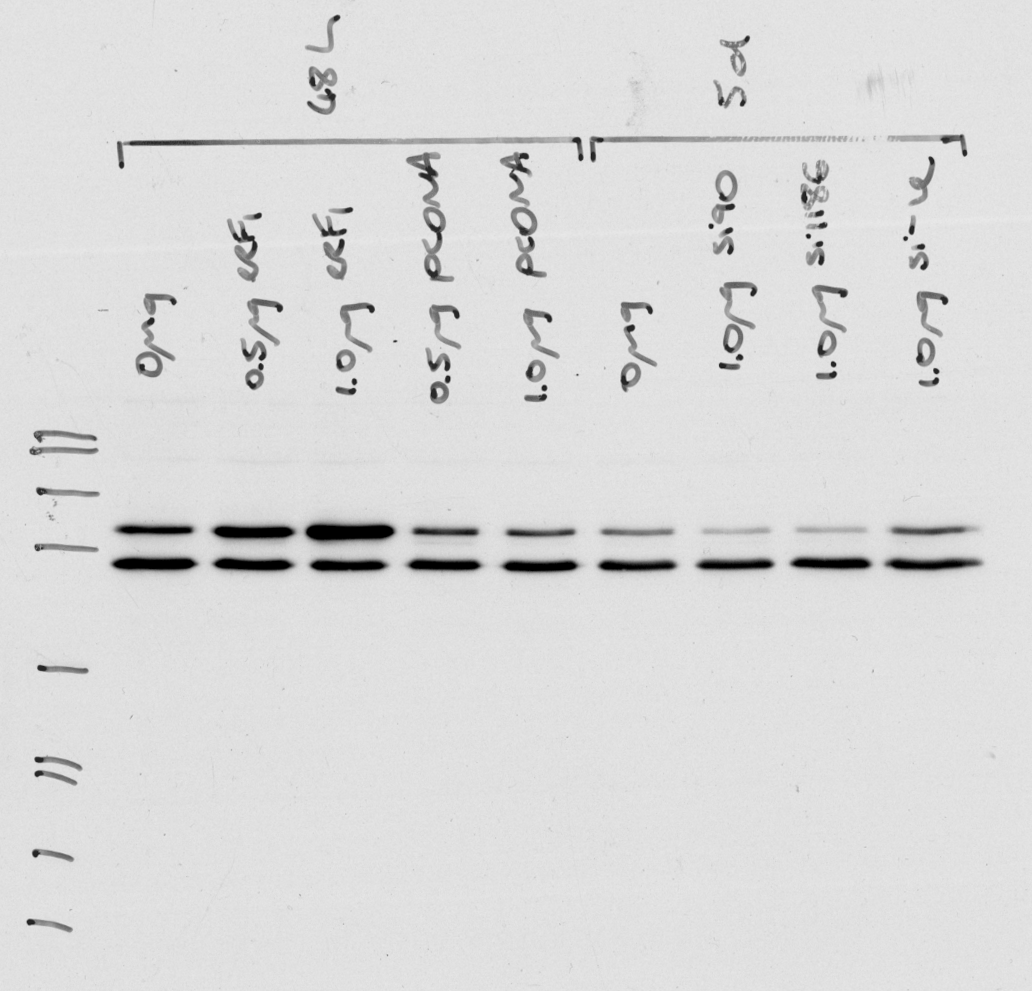

Supplement: S1 Fig — From left to right in Fig. 4B, bands from lanes ‘5d 1.0 μg si-ve’, ‘5d 1.0 μg si90’, and ‘5d 0 μg’ are shown. The leftmost lane contains markers corresponding proteins of, from top to bottom, 116.2 kDa, 97.4 kDa, 66.2 kDa, 45 kDa, 31 kDa, 21.5 kDa, 14.4 kDa, and 6.5 kDa. The 21.5 kDa molecular weight standard was marked as a doublet band in this transfer. (TIF) [file pone.0122176.s001.tif]

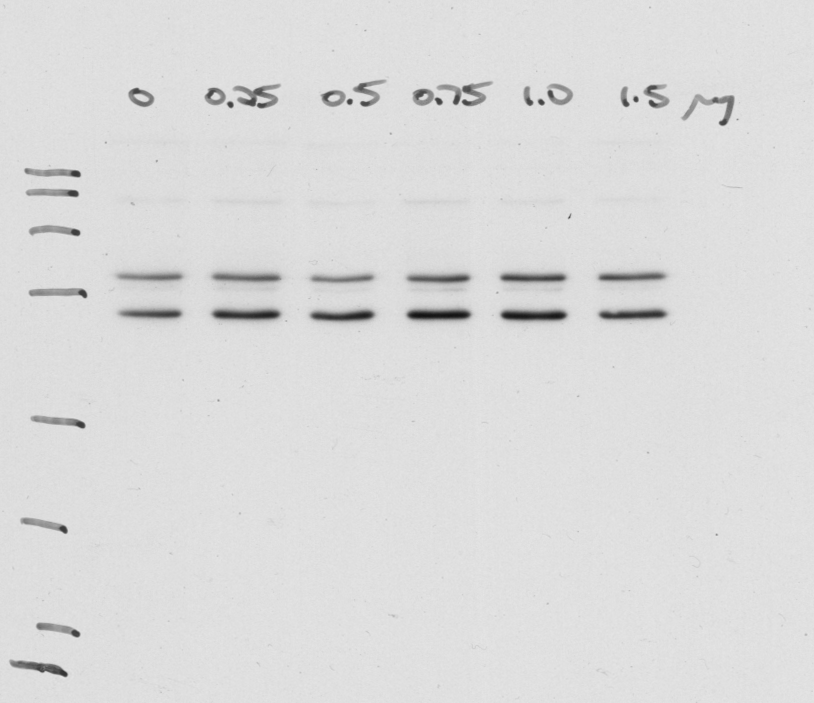

Supplement: S2 Fig — Bands from lanes ‘0’ and ‘1.0’ are shown in Fig. 5B as the leftmost ‘None’ and ‘Empty vector’ boxes, respectively. The leftmost lane contains markers corresponding to molecular weight standards of, from top to bottom, 116.2 kDa, 97.4 kDa, 66.2 kDa, 45 kDa, 31 kDa, 21.5 kDa, and 14.4 kDa. The last marker likely represents the bromophenol blue dye front. (TIF) [file pone.0122176.s002.tif]

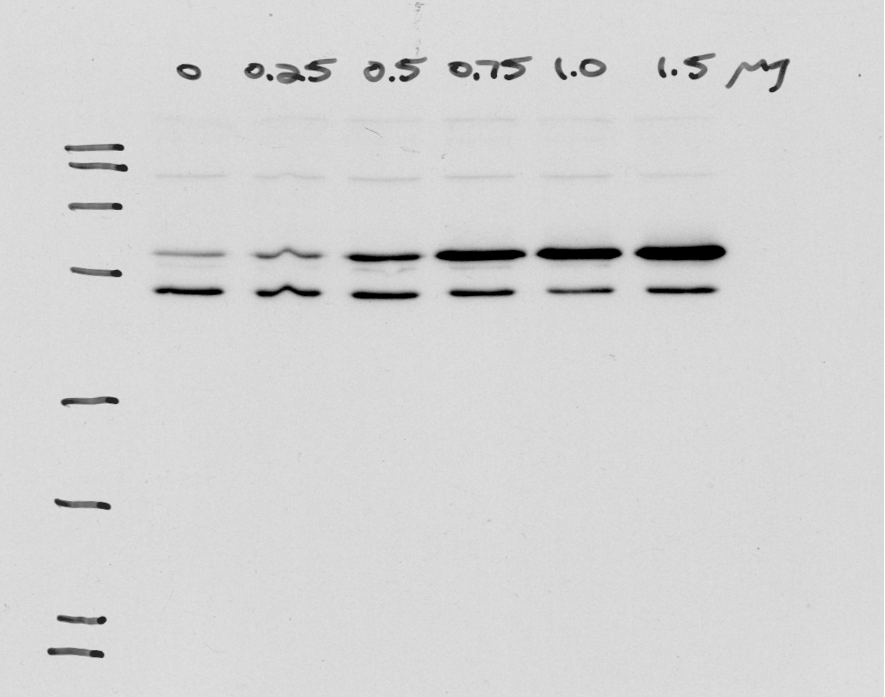

Supplement: S3 Fig — Bands from lanes ‘0’ and ‘1.0’ are shown in Fig. 5B as the rightmost ‘None’ and ‘eRF1’ boxes, respectively. Markers are the same as for S2 Fig. (TIF) [file pone.0122176.s003.tif]
