# Supplementary material for: The Highly Conserved Codon following the Slippery Sequence Supports −1 Frameshift Efficiency at the HIV-1 Frameshift Site
Source: PLoS One. 2015 Mar 25;10(3):e0122176. doi: 10.1371/journal.pone.0122176 (PMC4373837; doi:10.1371/journal.pone.0122176)
Supplement: S3 File — (DOCX) [file pone.0122176.s006.docx]

Output from a script looking at HIV genomes downloaded from <http://www.hiv.lanl.gov/> on 2012-11-28. Some of the checks presented here are internal controls.

4675 of 4675 total records are unique

3547 of 4675 records meet the LANL quality criteria

13 quality records have no aligned and intact slippery sequence, leaving 3534 usable records

3345 of 3534 records have a GGG intercodon (94.65 percent). There are 130 GGA and 50 GGC total.

99.75 percent of intercodons code for glycine, 0 total are GGR (i.e. annotated as "GGR" such in the input file), 4 total are AGG, 4 GAG, and 1 CGG

In conclusion, of 3534 intercodons, 3525 code for glycine, and 9 do not. This comes to 3534 total.
